# The Spindle Pole Bodies Facilitate Nuclear Envelope Division during Closed Mitosis in Fission Yeast

**DOI:** 10.1371/journal.pbio.0050170

**Published:** 2007-06-19

**Authors:** Liling Zheng, Cindi Schwartz, Valentin Magidson, Alexey Khodjakov, Snezhana Oliferenko

**Affiliations:** 1 Cell Dynamics Group, Temasek Life Sciences Laboratory, Singapore; 2 Department of Biological Sciences, National University of Singapore, Singapore; 3 The Boulder Laboratory for 3-D Electron Microscopy of Cells, Department of Molecular, Cellular and Developmental Biology, University of Colorado, Boulder, Colorado, United States of America; 4 Wadsworth Center, Albany, New York, United States of America; Dana-Farber Cancer Institute, United States of America

## Abstract

Many organisms divide chromosomes within the confines of the nuclear envelope (NE) in a process known as closed mitosis. Thus, they must ensure coordination between segregation of the genetic material and division of the NE itself. Although many years of work have led to a reasonably clear understanding of mitotic spindle function in chromosome segregation, the NE division mechanism remains obscure. Here, we show that fission yeast cells overexpressing the transforming acid coiled coil (TACC)-related protein, Mia1p/Alp7p, failed to separate the spindle pole bodies (SPBs) at the onset of mitosis, but could assemble acentrosomal bipolar and antiparallel spindle structures. Most of these cells arrested in anaphase with fully extended spindles and nonsegregated chromosomes. Spindle poles that lacked the SPBs did not lead the division of the NE during spindle elongation, but deformed it, trapping the chromosomes within. When the SPBs were severed by laser microsurgery in wild-type cells, we observed analogous deformations of the NE by elongating spindle remnants, resulting in NE division failure. Analysis of *dis1*Δ cells that elongate spindles despite unattached kinetochores indicated that the SPBs were required for maintaining nuclear shape at anaphase onset. Strikingly, when the NE was disassembled by utilizing a temperature-sensitive allele of the Ran GEF, Pim1p, the abnormal spindles induced by Mia1p overexpression were capable of segregating sister chromatids to daughter cells, suggesting that the failure to divide the NE prevents chromosome partitioning. Our results imply that the SPBs preclude deformation of the NE during spindle elongation and thus serve as specialized structures enabling nuclear division during closed mitosis in fission yeast.

## Author Summary

Chromosomes replicate in the nucleus, which is surrounded by the double-layered nuclear envelope (NE). During so-called open mitosis, the NE breaks down to allow spindle microtubules (which do the work of pulling the replicated chromosomes apart) access to the nuclear interior. Alternatively, in closed mitosis, many species assemble spindles inside the nucleus and segregate daughter genomes within the NE. To see how cells coordinate chromosome segregation with NE division in closed mitosis, we looked at the fission yeast Schizosaccharomyces pombe in which spindle poles are anchored at the spindle pole bodies (SPBs), specialized organelles inserted into the NE. We used two experimental manipulations to cause cells to assemble unusual spindles without the proper pole-located SPBs. These spindles deformed the NE and failed to segregate chromosomes. However, spindles lacking the SPBs were capable of segregating chromosomes if the NE was disassembled. We hypothesize that the SPBs have evolved as specialized structures preventing NE deformation during spindle elongation during closed mitosis.

## Abstract 3

Spindle pole bodies allow nuclear division during mitosis, when there is no nuclear envelope breakdown in fission yeast.

## Introduction

The nuclear envelope (NE) delineates the boundary of the nucleus in eukaryotic cells and is composed of two membranes enclosing a luminal space. Because chromosomes are located in the nucleus, cells must enable the access of spindle microtubules to the nucleoplasm in order to segregate the daughter genomes during mitosis. Cells undergoing open mitosis achieve this by breaking down the NE in prophase and reassembling it again in telophase. Alternatively, a number of species segregate chromosomes within the nucleus in the process known as closed mitosis.

Like most fungi, the fission yeast Schizosaccharomyces pombe elaborates the bipolar intranuclear mitotic spindle with the minus ends of microtubules anchored at specialized microtubule-organizing organelles, the spindle pole bodies (SPBs). The SPBs are functionally analogous to metazoan centrosomes and undergo a duplication and separation cycle generally correlated with the cell division cycle. The SPB of S. pombe spends most of interphase in the cytoplasm, adjacent to the NE [1,2]. However, it appears to be securely anchored at the outer NE as suggested by live-imaging studies of the SPB and the NE behavior in interphase [3,4]. As the SPB duplicates and matures, daughter SPBs remain connected by a bridge. Upon mitotic entry, a portion of the NE underlying the SPB pair invaginates, forming an opening (fenestra), and the SPBs settle into it. Each SPB initiates intranuclear microtubules, and they eventually separate and move to opposite sides of the nucleus while enclosed in their individual fenestrae that are somewhat larger than the SPBs themselves. In anaphase, the fenestrae contract to become about the same size as the SPBs. Late in mitosis, the fenestrae close, and the SPBs are extruded back into the cytoplasm [1]. These observations may indicate the existence of specialized and cell cycle–specific structures that function to anchor the SPB at the NE. Interestingly, the NE adjacent to the SPB itself shows some degree of specialization, including local thickening of the NE and presence of electron-dense material between the SPB and the NE [1,5]. Also, γ-tubulin material localizes to the dark-staining region underlying the SPB in interphase [1]. Thus, it seems that there is a complex of molecular associations that defines a structure, including both the SPB and an adjacent portion of the NE. At least one membrane-spanning protein, Cut11p, is known to participate in SPB anchorage during early mitosis [6].

An interesting corollary of closed mitosis is the requirement for regulated NE division in order to prevent chromosome entanglement during spindle elongation and to ensure proper NE and endoplasmic reticulum (ER) inheritance. The elongating spindle is a stiff structure that is capable of exerting considerable force on the NE. For instance, assembly of microtubule polymers within liposomes induces striking shape changes, indicating that microtubules are capable of deforming the cellular membranes [7,8]. The NE of S. pombe is a double-layered protein-containing structure that is likely more rigid than artificial phospholipid membranes. However, the NE is still capable of undergoing severe deformations due to forces exerted by microtubules, as suggested by three-dimensional reconstructions of monopolar spindles in *cut11* ^ts^ mutant cells [6] and live NE imaging during the process of spindle assembly (unpublished data) or in interphase [3]. Notably, the NE behaves in a radically different manner during mitosis, when it follows an ordered progression of shape changes resulting in accurate division. The NE remains relatively spherical throughout metaphase and anaphase A. As cells proceed to anaphase B, the NE first elongates to become an ellipse with spindle minus ends located at its poles. It eventually assumes a dumbbell shape, after which it undergoes fission resulting in the formation of two daughter nuclei.

Unlike microtubules polymerizing within liposomes [7,8], the elongating mitotic spindle contacts the membrane at specific sites, including the SPBs and, likely, the underlying specialized section of the NE. It is thus possible that the presence of these rigid structures could allow the NE to sustain the pushing forces produced by the elongating spindle. This way, the SPB could be considered not only a microtubule-organizing center, but also a supporting structural feature allowing NE division during closed mitosis.

Here, we describe experimental evidence supporting this hypothesis. While investigating mitotic functions of the transforming acid coiled coil (TACC)-related protein, Mia1p, in fission yeast, we found that cells overexpressing Mia1p failed to separate the SPBs, but frequently assembled elongating bipolar spindles. Under these circumstances, spindle poles deformed the NE, and NE division failed. Chromosome segregation was often unsuccessful, and DNA masses were confined within the undivided NE. Laser microsurgery in wild-type cells together with experiments on *dis1*Δ cells that exhibit faulty microtubule/kinetochore attachment, but normal spindle elongation, confirmed that the pole-located SPBs were required for NE division. Strikingly, genetic ablation of the NE during mitosis in Mia1p-overexpressing cells allowed sister chromatid segregation by acentrosomal spindles.

## Results

### Cells Overexpressing Mia1p Arrest in Mitosis, with Fully Elongated Spindles and Nonsegregated Chromosomes

**Figure 1:**
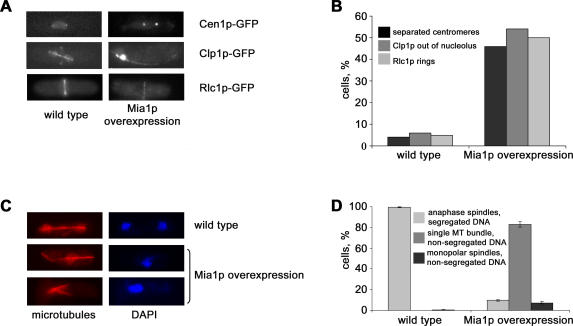
Fission Yeast Cells Overexpressing Mia1p Arrest in Mitosis with Fully Extended Spindles (A) The centromere markers, Cen1-GFP, Clp1-GFP, and Rlc1p-GFP, exhibit late mitotic localization in cells overexpressing Mia1p. Shown are single maximum-intensity reconstructions of live cells. (B) Graph quantifying the proportion of cells exhibiting mitotic localization of marker proteins (*n* = 100). (C) Immunofluorescence images of wild-type and Mia1p-overexpressing cells using anti–α-tubulin antibodies and the DNA dye, DAPI. (D) Graph quantifying the proportion of mitotic cells exhibiting wild-type or abnormal spindle architecture (*n* = 300). MT, microtubule.

Previous experiments suggested that the fission yeast TACC-related protein, Mia1p, is required for organizing and anchoring microtubule minus ends in interphase and for proper spindle function during mitosis [4,9,10]. To further our understanding of Mia1p function, we created S. pombe cells carrying a pREP1-based plasmid in which the *mia1*+ open reading frame was expressed under the control of the inducible *nmt1* promoter [11]. We found that overexpression of Mia1p in wild-type cells resulted in severe lethality (Figure S1, see also [10]). Overexpression of Mia1p in strains carrying various green fluorescent protein (GFP)-tagged cell cycle protein markers revealed that cells arrested during late mitosis. First, the centromere I marker, Cen1-GFP [12], was often found in two distinct and well-separated dots, indicating that anaphase promoting complex (APC) activation occurred and chromosome cohesion was lost (Figure 1A and 1B). We confirmed this by examining the localization of the general centromere/kinetochore marker, Mis6p-GFP [13], upon Mia1p overexpression. Mis6p-GFP labeled either two distinct kinetochore clusters, similar to wild-type control, or localized to six closely positioned dots, likely representing nonsegregated anaphase kinetochores (Figure S2). We also found that the Cdc14p-like phosphatase, Clp1p [14,15], localized to intranuclear spindles in the majority of cells, consistent with their mitotic state (Figure 1A and 1B). Moreover, most Mia1p-overexpressing cells exhibited actomyosin division rings, as judged by localization of the myosin II light chain, Rlc1p-GFP [16] (Figure 1A and 1B). Because the mitotic checkpoint protein, Mad2p-GFP [17], largely localized to the NE, it appeared that most Mia1p-overexpressing cells overcame the spindle assembly checkpoint and entered anaphase (Figure S3) (even though approximately 12% of cells exhibited Mad2p-GFP on kinetochores, indicative of some aspect of spindle malfunction). The component of the septation initiation network, Cdc7p-GFP [18], localized to a single SPB, both in control and Mia1p-overexpressing cells (unpublished data).

We performed immunofluorescence analyses of wild-type and Mia1p-overexpressing cells using anti–α-tubulin antibodies and the DNA dye, DAPI. All anaphase spindles in wild-type cells corresponded to two DNA masses located at the spindle poles, indicative of normal mitotic function (Figure 1C and 1D). Cells overexpressing Mia1p exhibited mitotic phenotypes that could be roughly divided into three groups: 10% of cells showed a wild-type morphology with respect to chromosome segregation; 7% of cells exhibited aster-like monopolar spindles and highly condensed chromosomes, and the rest displayed extended anaphase spindles and nonsegregated DNA masses (Figure 1C and 1D).

Taken together, our data indicated that Mia1p-overexpressing cells were predominantly arrested in anaphase, with fully extended spindles and nonsegregated chromosomes.

### The SPBs Are Displaced from Spindle Poles, and Nuclear Envelope Division Fails in Mia1p-Overexpressing Cells

**Figure 2:**
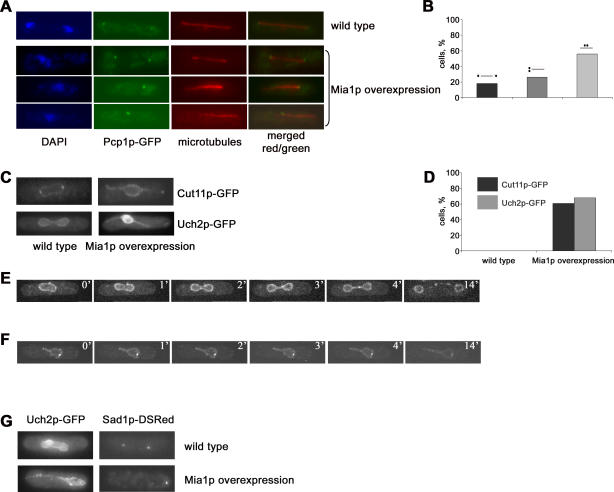
The SPBs Are Displaced from the Spindle Poles, and NE Division Fails in Cells Overexpressing Mia1p (A) Immunofluorescence images of wild-type and Mia1p-overexpressing cells using anti–α-tubulin antibodies, Pcp1p-GFP, and the DNA dye, DAPI. (B) Graph quantifying the proportion of mitotic Mia1p-overexpressing cells exhibiting normal or aberrant spindle architecture (*n* = 100). Figures over each bar schematically represent position of the SPBs (black dots) with respect to the spindle (grey line). (C) Mia1p overexpression induces panhandle-shaped deformations of the NE. Shown are single maximum-intensity reconstructions of live wild-type and Mia1p-overexpressing cells containing the NE marker proteins Cut11p-GFP and Uch2p-GFP. (D) Graph quantifying the proportion of cells exhibiting the panhandle-shaped NE deformations (*n* = 100). (E) Time-lapse sequence of Cut11p-GFP–expressing wild-type cells undergoing mitosis. Numbers refer to the time, in minutes. (F) Time-lapse sequence of Mia1p-overexpressing Cut11p-GFP cells undergoing mitosis. Note an elongating protrusion of the NE and the SPB pair on the opposite side of the nucleus. Numbers refer to the time, in minutes. (G) The SPBs largely localize away from the NE protrusion tips in cells overexpressing Mia1p. Note that in wild-type cells, the SPBs seem to lead the NE division. Shown are single maximum-intensity reconstructions of live wild-type and Mia1p-overexpressing cells containing the NE marker protein, Uch2p-GFP, and the SPB protein, Sad1p-DSRed.

The SPBs are thought to be required for mitotic spindle assembly and function in fungi, and are always found at the spindle poles (Figure 2A, wild type). We overexpressed Mia1p in S. pombe strains carrying the core SPB marker, Pcp1p-GFP or the peripheral SPB protein, Sad1p-GFP. Strikingly, we found that only 20% of Mia1p-overexpressing Pcp1p-GFP anaphase cells exhibited spindles with pole-located SPBs. Nonseparated SPB pairs located at one, but not the other, spindle pole were found in 25% of cells, whereas the rest of Mia1p-overexpressing cells exhibited duplicated SPBs that were completely mispositioned with respect to either spindle pole (Figure 2A and 2B). Overexpression of Mia1p in Sad1p-GFP cells confirmed that while the SPBs were duplicated and spindles elongated, the SPBs were often displaced from the spindle poles (Figure S4).

Microscopy analyses of the NE markers, Cut11p-GFP [6] and Uch2p-GFP [19], suggested that the extending intranuclear spindles often deformed the NE, with most cells exhibiting striking panhandle-shaped protrusions with chromosomes “trapped” in the central compartment (Figure 2C and 2D). To visualize NE dynamics, we performed time-lapse analyses of Cut11-GFP behavior in wild-type cells and cells overexpressing Mia1p. In the wild type, the NE went through dramatic changes after the onset of anaphase B. As the spindle elongated, the NE assumed an ovoid shape with the SPBs positioned at its leading edges. The dividing nucleus eventually underwent medial constriction. Finally, the remaining link between the two daughter nuclei was resolved (Figure 2E and Video S1). By contrast, in cells overexpressing Mia1p, we observed the appearance and extension of the NE protrusions at one (Figure 2F and Video S2) or both sides of the nucleus, and eventual failure of nuclear division.

Microscopy analysis of Mia1p-overexpressing Uch2p-GFP Sad1p-DSRed cells revealed that the nonseparated SPB pairs were usually found at the base of the protrusions or at the opposite side of the nucleus (Figure 2G).

**Figure 3:**
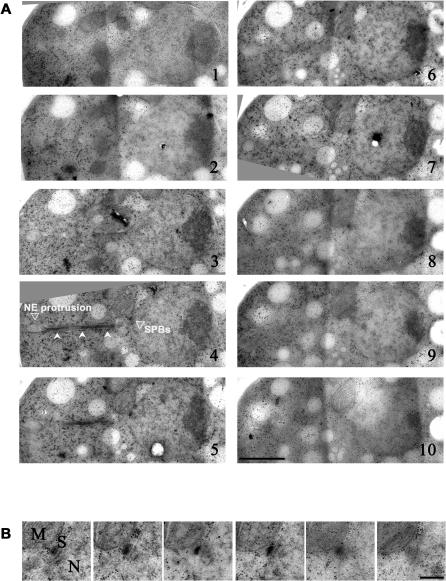
The SPBs Are Duplicated but Not Separated in Cells Overexpressing Mia1p (A) Electron microscopy images of serial sections of a Mia1p-overexpressing cell showing the abnormal panhandle-shaped protrusion of the NE. Note that microtubules extend throughout the protrusion (indicated by indented arrowheads, panel 4). The SPB pair is located at the base of protrusion. The NE protrusion and position of the SPBs are indicated on panel 4. Scale bar represents 1 μm. (B) Higher magnification image of the duplicated SPB pair. Mitochondrion is labeled as M, nucleus as N. Scale bar represents 0.2 μm.

To gain insight into the intranuclear architecture upon overexpression of Mia1p, we performed electron microscopy serial-section analyses of rapidly frozen and freeze-substituted cell samples, followed by three-dimensional image reconstruction [20–22]. We confirmed that the SPBs were duplicated, but not separated (Figure 3A and 3B, and Video S3). In the example shown, the spindle is positioned between the SPB pair at the base of the protrusion and the projection tip. The free end of the spindle deforms the double-layered NE (Figure 3A and Video S3).

Thus, we concluded that (1) the SPBs were duplicated, but not separated; (2) the SPBs were often disassociated from spindle poles; and (3) the SPB-free, or acentrosomal, spindle poles deformed the NE during spindle extension in Mia1p-overexpressing cells.

### Spindles in Mia1p-Overexpressing Cells Are Bipolar and Require the Microtubule-Crosslinking Protein, Ase1p, for Their Assembly

**Figure 4:**
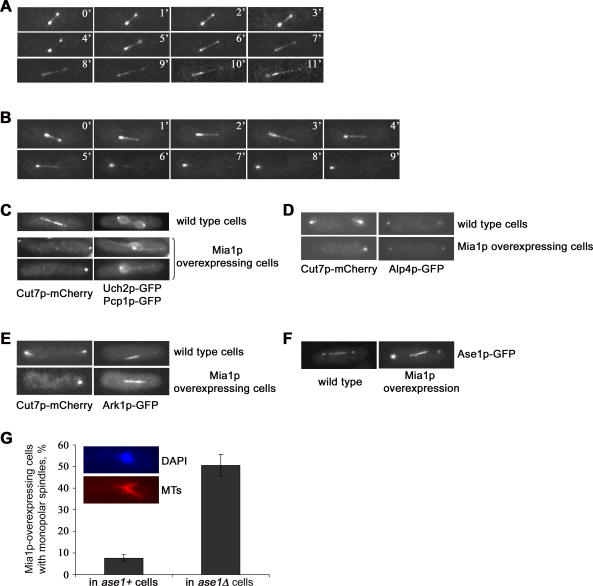
Spindle Structures in Mia1p-Overexpressing Cells Are Bipolar and Antiparallel (A) Time-lapse sequence of Cut7p-GFP–expressing wild-type cells undergoing spindle elongation. Numbers refer to the time, in minutes. (B) Time-lapse sequence of Mia1p-overexpressing Cut7p-GFP cells undergoing mitosis. Note that Cut7p-GFP clearly localizes to both poles early in anaphase and is progressively depleted from one of them as the cell progresses through mitosis. The midzone localization is similar to wild type. Shown are single maximum-intensity reconstructions of *z*-stacks. Numbers refer to the time, in minutes. (C) Cut7p-mCherry is enriched on acentrosomal spindle poles as judged by double fluorescence microscopy analysis with the SPB core marker, Pcp1p-GFP. Shown are single maximum-intensity reconstructions of *z*-stacks. (D) The γ-TuRC marker, Alp4p-GFP, is present on acentrosomal poles in Mia1p-overexpressing cells, where it co-localizes with Cut7p-mCherry. Shown are single maximum-intensity reconstructions of *z*-stacks. (E) The chromosomal passenger complex protein, Ark1p-GFP, defines the spindle midzone in acentrosomal spindles. Shown are single maximum-intensity reconstructions of *z*-stacks. (F) Ase1p-GFP localizes to the spindle poles and spindle midzone in wild-type and Mia1p-overexpressing cells. Shown are single maximum-intensity reconstructions of *z*-stacks. (G) Graph quantifying the proportion of cells exhibiting monopolar spindles upon Mia1p overexpressing in wild-type and *ase1*Δ genetic backgrounds (*n* = 300). Inset, immunofluorescence image of the monopolar spindle in a Mia1p-overexpressing *ase1*Δ cell using anti–α-tubulin antibodies and the DNA dye, DAPI. MTs, microtubules.

Given that the overexpression of Mia1p induced assembly of elongating spindles even though SPBs were not separated, we were interested in determining the polarity of microtubules in these structures. The BimC-related tetrameric kinesin, Cut7p, is essential for spindle formation and exhibits a complex cell cycle–specific pattern of localization [23,24]. In metaphase wild-type cells, Cut7p-GFP was concentrated at spindle poles and localized along the entire spindle length. As the cell proceeded into anaphase, Cut7p-GFP mostly redistributed along the spindle to eventually concentrate in the broad midzone area, consistent with its function in spindle elongation. It also exhibited a weaker association with the SPBs (Figure 4A, ten out of ten cells). Interestingly, Cut7p-GFP exhibited a largely bipolar localization in Mia1p-overexpressing cells, somewhat similar to wild type. Time-lapse analyses of elongating spindles indicated that they were clearly bipolar until early anaphase B (eight out of 11 cells). As spindles elongated, Cut7p-GFP redistributed to the spindle midzone and was eventually almost completely depleted from one of the spindle poles while being enriched on the other one (Figure 4B). Fluorescence microscopy analyses utilizing Cut7p-mCherry, Uch2p-GFP, and Pcp1p-GFP marker proteins indicated that Cut7p was enriched on acentrosomal spindle poles in cells overexpressing Mia1p (93% of cells, *n* = 100, see Figure 4C). Thus, even though the Cut7p-GFP localization was not symmetric in late anaphase spindles, it defined the spindle midzone, suggesting that orientation of microtubules in Mia1p-induced spindles was antiparallel with plus ends in the center of the spindle, similar to wild type.

Consistently, the γ-tubulin ring complex (γ-TuRC) component, Alp4p-GFP [25], concentrated at both spindle poles in the control and Mia1p-overexpressing cells, as judged by double fluorescence microscopy analyses using Cut7p-mCherry as a spindle marker (Figure 4D). Also, although in control cells, the Alp4p-GFP signal always coincided with the SPB marker, Sad1p-DSRed, we observed the presence of Alp4p-GFP-positive, Sad1p-DSRed-negative structures upon Mia1p overexpression indicative of γ-TuRC–containing acentrosomal poles (Figure S5).

We further evaluated spindle polarity by overexpressing Mia1p in cells containing spindle midzone markers. We found that the member of the chromosomal passenger complex aurora kinase, Ark1p-GFP [26], localized to kinetochores in metaphase (~1% of cells) and to the spindle midzone during anaphase (~5% of cells) in control cells. Ark1p-GFP defined the spindle midzone in 43% of Mia1p-overexpressing cells, and we found it localizing to kinetochores in 15% of cells (Figure 4E).

The microtubule-binding protein Ase1p is another spindle midzone marker that has been suggested to contribute to proper midzone formation and the structural integrity of the bipolar spindle [27,28]. Although Ase1p is not essential, spindles in *ase1*Δ cells tend to collapse, but maintain bipolarity, and occasionally break during anaphase elongation [27]. Ase1p-GFP localized to the region of the spindle midzone and spindle poles in Mia1p-overexpressing cells (Figure 4F). Strikingly, we found that Ase1p was required for the assembly of acentrosomal bipolar spindles upon Mia1p overexpression. The number of cells exhibiting typical aster-like monopolar spindles dramatically increased when Mia1p was overexpressed in the *ase1*Δ genetic background (Figure 4G). Control cells lacking Ase1p, but not overexpressing Mia1p, exhibited a very low incidence of monopolar spindles (less than 0.5%).

Thus, we concluded that Mia1p-overexpressing cells could assemble bipolar and antiparallel spindle structures despite failure of SPB separation.

### Elongation of Acentrosomal Spindles Results in Protruding Deformations of the Nuclear Envelope and Failure of Nuclear Division

**Figure 5:**
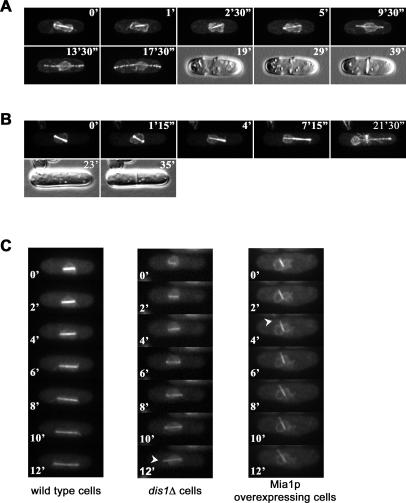
NE Division Fails When the SPBs Are Not Positioned at Elongating Spindle Poles (A) Time-lapse sequence of a Cut11p-GFP α-tubulin-GFP–expressing wild-type cell when both SPBs were severed from the elongating anaphase spindle by laser microsurgery. (B) Time-lapse sequence of a Cut11p-GFP α-tubulin-GFP–expressing wild-type cell when one SPB was cut off the spindle. Note that the NE is forming the panhandle-shaped protrusions at the sites of contact with the elongating spindle remnants. DIC images at the end of the sequence show normally proceeding septation. Shown are single maximum-intensity reconstructions of *z*-stacks. Numbers refer to the time, in minutes and seconds. (C) Time-lapse fluorescence microscopy analyses of the NE and spindle dynamics in Uch2p-GFP α-tubulin-GFP–expressing wild-type, *dis1*Δ, and Mia1p-overexpressing cells. Formation of membrane tethers in *dis1*Δ and Mia1p-overexpressing cells is indicated by arrowheads. Shown are single maximum-intensity reconstructions of *z*-stacks. Numbers refer to the time, in minutes.

Spindle structures assembled in cells overexpressing Mia1p failed to segregate chromosomes and to ensure NE division. A striking feature of Mia1p-induced spindles was a conspicuous absence of the SPBs at one or both spindle poles. We sought to determine whether the SPBs were in fact required for providing a structural rigidity that could allow spindle poles to push against the NE without deforming it. To test this hypothesis, we severed the SPBs from early anaphase spindles in α-tubulin-GFP Cut11p-GFP (Figure 5A and 5B), and in α-tubulin-GFP Uch2p-GFP (unpublished data) expressing wild-type cells using laser microsurgery [29]. When both SPBs were cut off, spindles continued elongation due to sliding forces produced in the spindle midzone [29]. These spindle remnants readily formed panhandle-shaped protrusions at opposite sides of the nucleus (Figure 5A), evocative of the phenotype observed in Mia1p-overexpressing cells (Figure 2C), and similar to what has been previously noted [29]. When the SPB was severed from one spindle pole, spindles deformed the NE at this location, whereas no NE deformation was observed at the opposite spindle pole still tethered to the other SPB (Figure 5B).

It is possible that the presence of either the SPB or of chromosomes at the poles, or both, is required for maintaining shape of the nucleus during anaphase movement. To distinguish between these possibilities, we performed time-lapse analyses of cells lacking the MAP215-related protein, Dis1p, and expressing the NE marker, Uch2p-GFP, and the microtubule marker, α-tubulin-GFP. It has previously been shown that at the restrictive temperature of 20 °C, Dis1p is required for kinetochore/microtubule attachment although the spindle elongates to the full extent [30,31]. We found that initial elongation of the NE proceeded normally in *dis1*Δ cells as compared to wild-type control, and eventually, nuclei of both cell types assumed an ovoid shape (Figure 5C). However, as the spindle in *dis1*Δ cells elongated further, the NE abruptly collapsed, resulting in the formation of the panhandle-shaped protrusion (Figure 5C). We then performed imaging of Mia1p-overexpressing cells that also contained Uch2p-GFP α-tubulin-GFP during the same stages of mitosis. In the presence of overexpressed Mia1p, constitutive expression of α-tubulin-GFP failed to generate fluorescent bipolar spindles (unpublished data). Therefore, we simultaneously induced expression of Mia1p from the *nmt1* promoter and α-tubulin-GFP from the weak *nmt81* promoter. We found that although there was still a considerable increase in the number of cells with monopolar spindles, we could observe few cells with properly assembled elongating spindles. The NE in these cells was abnormally deformed immediately after spindle elongation, without going through the initial expansion and elongation phase (Figure 5C).

Based on these experiments, we concluded that the SPBs positioned at spindle poles were essential for NE division and that successful segregation of the NE was sustained by keeping daughter chromosomes attached to the SPBs throughout anaphase B.

### Genetic Ablation of the Nuclear Envelope Allows Chromosome Segregation in Mia1p-Overexpressing Cells

**Figure 6:**
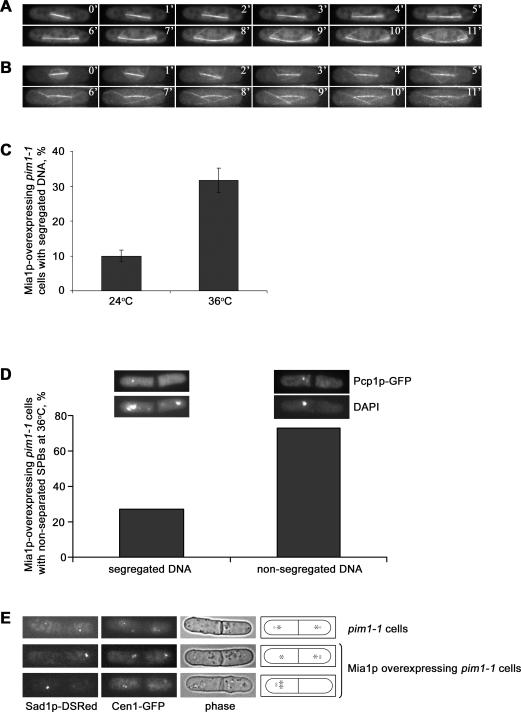
DNA Segregation Occurs in Mia1p-Overexpressing Cells When the NE Is Fragmented in Mitotic Cells Harboring the *pim1–1* Mutation (A) Time-lapse sequence of an Uch2p-GFP α-tubulin-GFP–expressing *pim1–1* cell undergoing anaphase at the permissive temperature of 24 °C. (B) Time-lapse sequence of an Uch2p-GFP α-tubulin-GFP–expressing *pim1–1* ^ts^ cell undergoing anaphase at the restrictive temperature of 36 °C. Cells were shifted to the restrictive temperature 3 h prior to imaging to allow inactivation of Pim1p protein. Note that the NE is fragmented shortly after anaphase B onset. Shown are single maximum-intensity reconstructions of *z*-stacks. Numbers refer to the time, in minutes. (C) Graph quantifying the proportion of binucleate Pcp1p-GFP Uch2p-GFP *pim1–1* cells overexpressing Mia1p at 24 °C and 36 °C (*n* = 300). (D) Graph quantifying the proportion of uninucleate and binucleate Mia1p-overexpressing Pcp1p-GFP Uch2p-GFP *pim1–1* cells that do not separate the SPBs at 36 °C (*n* = 100). Insets, examples of the scored phenotypes. Epifluorescence images of fixed Mia1p-overexpressing *pim1–1* cells containing the core SPB marker, Pcp1p-GFP, and the outer NE marker, Uch2p-GFP, at 36 °C. DNA is stained with DAPI. The NE is fragmented. Shown are single maximum-intensity reconstructions of *z*-stacks. (E) Epifluorescence images of Cen1-GFP Sad1-DSRed–containing *pim1–1* cells, with and without Mia1p overexpression. Shown are single maximum-intensity reconstructions of *z*-stacks. Cartoons outline the phenotypes scored. Circle indicates the SPB; star indicates the centromere I.

As shown above, Mia1p overexpression is lethal, largely due to the failure in nuclear division and chromosome segregation. Because most Mia1p-overexpressing cells bypassed the spindle assembly checkpoint and arrested in anaphase, we wondered whether, in part, it was the NE division defect that interfered with chromosome segregation due to entrapment of DNA masses. Thus, we tested whether the Mia1p-induced spindles could segregate DNA at least at some frequency in an experimental situation in which cells underwent mitosis in the absence of the NE. The temperature-sensitive mutant in the Ran-GEF, *pim1–1,* has been reported to induce NE fragmentation upon passage through mitosis [32]. Time-lapse analyses of spindle and NE dynamics in α-tubulin-GFP Uch2p-GFP *pim1–1* cells at the permissive (Figure 6A and Video S4) and restrictive (Figure 6B and Video S5) temperatures suggested that lack of Pim1p function induced an irreversible NE fragmentation in early anaphase B. Spindles continued elongating (Figure 6B and Video S5), and cells eventually underwent cytokinesis, arresting at this point in the cell cycle ([32] and Figure 6D). We induced overexpression of Mia1p in *pim1–1* cells containing the integral SPB marker, Pcp1p-GFP, and the NE marker, Uch2p-GFP, at either permissive or restrictive temperature. Panhandle-shaped NE protrusions could be induced in *pim1–1* cells at 24 °C at a rate similar to control Uch2p-GFP cells (Figure S6). Only 10% of Pcp1p-GFP Uch2p-GFP *pim1–1* cells overexpressing Mia1p segregated DNA in two masses when the NE was intact (Figure 6C), consistent with the above experiments. When the NE was disassembled upon shifting cells to the restrictive temperature, the proportion of binucleate cells increased considerably (32%) (Figure 6C).

We found that few cells with spindles lacking the SPBs at least at one spindle pole exhibited two or more closely positioned DNA masses when the NE was intact (4% of cells, Figure S7). However, when the NE was fragmented, we observed an appearance of divided cells with DNA segregated to daughter compartments. Upon the shift to the restrictive temperature, 27% of cells exhibiting duplicated, but not separated, SPBs on one side of the septum showed presence of chromosomes in both daughter cells (*n* = 100, Figure 6D). We repeated this experiment using cells expressing the centromere I marker, Cen1-GFP, and the SPB marker, Sad1p-DSRed. We found that 32% of cells (*n* = 68) with acentrosomal spindles properly segregated sister chromatids when the NE was fragmented, suggesting that failure in NE division could hinder DNA segregation (Figure 6E).

Taken together, our results suggest that (1) overexpression of the TACC-related protein, Mia1p, results in the assembly of bipolar and moderately functional spindles that lack properly positioned SPBs; (2) one of the essential functions of the SPB in fission yeast could be to facilitate NE division during closed mitosis; and (3) failure to divide the NE could result in restriction of DNA masses movement and, ultimately, in a chromosome segregation defect.

## Discussion

In this study, we show that anaphase elongation of acentrosomal spindles during closed mitosis in fission yeast causes NE division failure and associated chromosome segregation defects.

Mia1p belongs to a family of microtubule-associated proteins named after the human transforming acid coiled coil protein, TACC, which has been implicated in cancer development (for review, see [33]). TACC proteins participate in spindle pole focusing and organization in metazoans [34,35]. Fission yeast cells lacking Mia1p exhibit a variety of microtubule defects throughout the cell cycle. Mitotic *mia1*Δ cells lack astral microtubules [9] and show signs of defective kinetochore/microtubule attachment [10]. During interphase, *mia1*Δ cells fail to establish and maintain properly organized microtubule arrays, likely due to a molecular defect in microtubule attachment to the original nucleation sites [4]. These functions correlate well with the Mia1p localization pattern. Indeed, Mia1p-GFP is mainly concentrated at the microtubule-organizing centers, though it is also found at plus ends of microtubules [4,9,10].

We found that cells overexpressing Mia1p were capable of organizing ectopic spindle poles and acentrosomal spindles. Although formation of intranuclear microtubule bundles in interphase S. pombe cells has previously been observed [36], overexpression of Mia1p led to assembly of mitotic spindles exhibiting normal microtubule arrangement (Figures 1 and 4). These spindles contained the γ-TuRC complexes and the BimC kinesin, Cut7p, at acentrosomal spindle poles, and exhibited a well-defined spindle midzone (Figure 4). Curiously, unlike the case of normal wild-type spindles, formation of these structures entirely depended on the presence of the microtubule-crosslinking protein, Ase1p (Figure 4G). Mia1p-GFP expressed at high levels localized to spindle poles, where it formed large fluorescent structures, and elsewhere in the cytosol (unpublished data). Interestingly, upon overexpression of Mia1p, Ase1p-GFP aggregated at the spindle poles in addition to its normal localization to the spindle midzone (Figure 4F). The γ-TuRC component, Alp4p-GFP, also localized to the acentrosomal spindle poles (Figure 4D and Figure S5), suggesting that they could indeed contain a host of microtubule-associated factors. The fact that some proteins required for proper spindle assembly might be sequestered to these abnormal structures could also explain the reason why the SPBs failed to separate in cells overexpressing Mia1p. TACC proteins contain extensive coiled coil regions and can likely form oligomers. This, in combination with their microtubule binding properties, could provide a framework for clustering and focusing of microtubule minus ends observed in vivo. We envisage that accumulation of the γ-TuRC complexes and microtubule-crosslinking proteins near the SPBs, followed by subsequent sliding off through the force produced by the growing spindle, could lead to the formation of acentrosomal spindle poles.

A particularly interesting phenotype of Mia1p overexpression was the formation of long, panhandle-shaped NE protrusions upon spindle elongation and the fact that NE division failure was reproducibly accompanied by DNA segregation defects. One possible explanation for such a phenotype could be the lack of microtubule/kinetochore attachment in these cells, which is not particularly surprising given that Mia1p normally functions in this process. We did observe an occurrence of trailing chromosomes in some cells overexpressing Mia1p when the SPBs delineated both spindle poles (for example, see Figure 2A). Alternatively, the presence of the SPB inserted into the specialized region of the NE could prevent the formation of membrane tethers during spindle elongation. We found that most acentrosomal spindle poles in Mia1p-overexpressing cells distorted the NE, whereas most poles associated with the SPBs did not exhibit this behavior (Figure 2). We also performed a series of laser microsurgery experiments in which we severed either one or both SPBs from the rest of the elongating anaphase spindle (Figure 5A and 5B). We found that, similar to Mia1p overexpression, the free spindle poles deformed the NE, and these cells failed to divide their nuclei despite normally timed septation (Figure 5A and 5B).

We sought to distinguish between the possibilities outlined above by following the NE dynamics in cells lacking the MAP215 protein, Dis1p, at the restrictive temperature of 20 °C. *dis1*Δ cells do not attach sister chromatids to the spindle microtubules, but proceed with spindle elongation [30,31]. Because spindles in *dis1*Δ cells contained the SPBs, we could assess relative contributions of chromosomes and SPBs to NE division. We found that initially, the NE in *dis1*Δ cells went through normal elongation and assumed the ovoid shape typical of wild-type nuclei (Figure 5C). However, during subsequent spindle elongation, the NE abruptly collapsed, suggesting that positioning of chromosomes at the poles was essential for maintaining the shape of the dividing nucleus (Figure 5C, indicated by an arrowhead). It is possible that once the membrane reservoir (see below) is exhausted during the initial elongation stage, a mass of chromosomes provides a physical block to the NE division. Contrary to the *dis1*Δ phenotype, spindles immediately produced nuclear membrane tethers in Mia1p-overexpressing cells (Figure 5C, emerging protrusion is indicated by an arrowhead), and nuclei did not assume the elliptical shape. We concluded that both the SPBs and the spindle pole–positioned chromosomes were essential for successful NE division.

The SPB of S. pombe undergoes striking changes in morphology and cellular localization during the cell cycle. Because the SPBs move in and out of the nuclear membrane, depending on the mitotic phase, this process likely relies on a tightly regulated membrane-anchoring system. It has been suggested that Cut11p, a transmembrane protein similar to budding yeast Ndc1p, might participate in SPB anchoring during mitosis. In cells lacking Cut11p function, the SPBs are frequently found floating in the nucleoplasm, in addition to the pronounced SPB separation defect [6]. Cut11p localizes to the nuclear pores throughout the cell cycle and is recruited to the SPBs at the onset of mitosis, which would be consistent with its proposed function in the SPB anchorage. Interestingly, at least one computer-generated reconstruction of a defective spindle in Cut11p-deficient cells shows an image very similar to spindles assembled in Mia1p-overexpressing cells. The spindle is bipolar, but only one spindle pole is connected to the SPB. There is no SPB at the other pole that forms a herniated protrusion on the NE [6].

However, the SPB localization of Cut11p is lost in mid-anaphase B despite the fact that the SPBs still appear to lead the nuclear movement ([6] and unpublished data). Thus, it is possible that there is an additional mechanism executing proper SPB tethering and NE reinforcement at the site of insertion. The integral NE protein and SPB component, Sad1p, could possibly function in this process [37]. Sad1p-related proteins from other organisms have been involved in anchoring centrosomes to the NE [38–40]. Also, the intranuclear system of Mlp filaments might be a good candidate for reinforcing the site of SPB attachment to the NE. Mlps are coiled coil filamentous proteins localized to the nucleoplasmic side of the NE [41]. Both budding yeast Mlp1p and Mlp2p localize to the NE crescent region underlying the SPB, with Mlp2 forming discernible foci. Interestingly, Mlp2p interacts with the core components of the SPB and is required for proper SPB assembly and function [42]. It is possible that a large structure such as the SPB has to be anchored into the underlying assembly of Mlp filaments. It is also possible that the Mlp assembly might structurally reinforce the NE in order to maintain the stiffness and the appropriate curvature of the membrane during closed mitosis. It will be of significant interest to address potential roles of Sad1p and Mlp proteins in NE division in future studies.

Panhandle-shaped protrusions of the nuclear membrane produced in our experiments can be compared to membrane tethers produced by microtubule polymerization or application of laser tweezers in artificial lipid vesicles [7,43]. Upon force application, the membrane develops regions of negative curvature and collapses into narrow tubes around the spindle. Interestingly, formation of these long tethers may suggest the existence of a functional membrane reservoir at this stage of the cell cycle [44]. We performed time-lapse analyses of wild-type cells (*n* = 7) expressing the NE marker, Uch2p-GFP, and α-tubulin-GFP, and measured the nuclear diameter at metaphase (3.76 ± 0.14 μm) and anaphase (3.12 ± 0.12 μm). Assuming nuclei to be spherical, the combined surface area of the two daughter nuclei is estimated to be approximately 1.37 times larger than that of the mother nucleus. This apparent increase in surface area could result from membrane contribution from the ER or unraveling of nuclear membrane folds. At the same time, it is conceivable that the nuclear envelope behaves like a rigid network of locally deformable components. Studies performed on the NE of mammalian cells suggest that it could be organized as a heterogeneous meshwork consisting of regular lattice domains and interconnecting filaments [45]. Under mechanical stress, the meshwork would expand resulting in vacancies of lamin A. Although fungal nuclear envelopes do not contain lamins, it is possible that other proteins underlying the inner membrane, for instance, Mlp filaments, could perform similar function in connecting the network.

We reasoned that should the undivided NE restrict the movement of chromosomes in cells overexpressing Mia1p, the removal of this obstacle could enable some degree of chromosome segregation through the action of acentrosomal spindles. Cells harboring a mutation *(pim1–1)* in a gene encoding for the RCC1-related protein, Pim1p, have been shown to undergo NE fragmentation upon passage through mitosis, and arrest with condensed DNA following hyperseptation ([32], see also Figure 6A and 6B, and Videos S4 and S5). We indeed found that a failure of DNA segregation in Mia1p-overexpressing cells was considerably relieved in the *pim1–1* genetic background (Figure 6C and 6D). Interestingly, a significant proportion of these cells could accurately segregate sister chromatids, suggesting that acentrosomal spindles were functional although less efficient than normal ones (Figure 6D). It is likely that a combination of factors contributes to the low efficiency of Mia1p-induced acentrosomal spindles in chromosome segregation. First, as outlined above, Mia1p overexpression could interfere with kinetochore attachment to spindle microtubules. Secondly, it is possible that attachment of microtubules to the SPB contributes to the increased accuracy of chromosome segregation, similar to what has been suggested for the case of centrosomes in animal cells [46].

There is an enormous structural diversity of spindles in eukaryotes [47,48]. Apart from the well-established open and closed mitosis categories, spindles can assemble as extra- or intranuclear ones [47]. Although in some organisms the NE remains intact, in others, it might partially break down [49,50] or be forcibly stripped off [51]. Similarly, various structural modifications throughout evolution resulted in the appearance of various types of SPBs. Here, we suggest that the highly structured SPB of fission yeast, and likely of other organisms in which chromosome segregation occurs strictly within the nuclear membrane, functions not only as a microtubule-organizing center, but also serves as a structural feature that enables NE division.

## Materials and Methods

### S. pombe strains, antibodies, reagents, and constructs.

S. pombe strains used in this study and their genotypes are listed in Table S1. EMM2 medium with suitable supplements was used for vegetative growth. For live-imaging experiments Mia1p overexpression was routinely induced for 18 h at 24 °C in EMM2 lacking thiamine. Genetic crosses and sporulation were performed on YPD agar plates. Plasmids were constructed using standard techniques of molecular cloning. The anti–α-tubulin antibody, TAT-1, was a gift from Dr. K. Gull.

### Immunofluorescence techniques.

Cells were fixed with 3.7% formaldehyde for 12 min and then spheroplasted using lysing enzymes and zymolyase in 1.2 M sorbitol in PBS. Permeabilization was performed in 1% Triton X-100 in PBS. Spheroplasts were washed with PBS. PBAL buffer (1 mM sodium azide, 1% BSA, 100 mM lysine hydrochloride, 50 μg/ml carbenicilin in PBS) was used for blocking and for incubation with primary and secondary antibodies. Imaging was done on a Zeiss Axiovert 200M microscope (Carl Zeiss, http://www.zeiss.com) with appropriate sets of filters, and images were generated using a CoolSnap camera (Photometrics, http://www.photomet.com) and Metamorph software package (Universal Imaging/Molecular Devices, http://www.moleculardevices.com). Image processing was done in Adobe Photoshop 7.0.1 (Adobe Systems, http://www.adobe.com).

### Time-lapse fluorescent microscopy.

Three-dimensional time-lapse images were generated on a Zeiss Axiovert 200M microscope equipped with CSU10 confocal optical scanner, 488-nm laser illumination source, and CoolSnap digital camera (Photometrics). Alternatively, we performed imaging in epifluorescence mode using a mercury lamp as an illumination source together with appropriate sets of filters, CoolSnap camera (Photometrics), and Uniblitz shutter driver (http://www.uniblitz.com) under the control of Metamorph software package (Universal Imaging). Typically, we acquired image stacks that consisted of five or six sections at 0.5-μm spacing. Imaging at 20 °C was performed using a homemade, cooled microscope housing.

### Laser microsurgery.

Laser microsurgery was conducted on a custom-assembled microscopy workstation centered around a Nikon TE2000-E2 microscope (Nikon Instruments, http://www.nikon.com). Laser pulses (532 nm for 8 ns) were generated by Q-switched Nd:YAG laser (Diva II; Thales Lasers, http://thales.nuxit.net) run at a 20-Hz repetition rate. Collimated laser beam was expanded to approximately 8 mm to fill the aperture of a 100× 1.4 NA PlanApo lens and delivered through an dedicated epi-port. Fluorescence images were recorded with a Cascade512B back-illuminated EM-CCD camera (Photometrics) attached to the left microscope port (100% transmission) in confocal mode (spinning disk confocal; Perkin-Elmer, http://www.perkinelmer.com) as three-dimensional datasets at 0.25-μm Z steps. Differential interference contrast (DIC) images were recorded with a CoolSnap CF CCD camera attached to the right port of the microscope (modified to 100% transmission). All light sources were shuttered by either fast mechanical shutters (Vincent Associates, http://www.uniblitz.com) or AOTF (Solamere Technology Group, http://www.solameretech.com) so that cells were exposed to light only during laser operations and/or image acquisition.

### Electron microscopy.

Cells were rapidly frozen by high-pressure freezing (BAL-TEC HPM-010; Technotrade International, http://www.technotradeinc.com/) and freeze-substituted at −90 °C in 2% glutaraldehyde plus 0.1% uranyl acetate in acetone for 96 h in an EM-AFS device (Leica, http://www.leica-microsystems.com). The cells were warmed for more than 3 h to −60 °C and then infiltrated with HM20 (Electron Microscopy Sciences, http://www.emsdiasum.com) resin over a period of 3 d. The cells were embedded under UV light at −60 °C in HM20 for 3 d and then warmed to room temperature over a 6-h period. Serial sections (60 nm) of embedded cells were picked up on Formvar-coated slot grids, stained with 1% aqueous uranyl acetate and lead citrate, and imaged in a Philips Tecnai TF20 FEG electron microscope (http://www.fei.com/) operating at 80 keV. Serial sections were aligned and modeled using the IMOD software package.

## Supporting Information

Figure S1 Overexpression of Mia1p in S. pombe Is Lethal Cells carrying either an empty pREP1 vector or the pREP1-based plasmid containing *mia1^+^* open reading frame (ORF) were grown overnight in EMM2 medium containing thiamine to the optical density at 595 nm (OD_595_) equal to 0.4. They were spotted onto EMM2 plates with or without thiamine at increasing 10× dilutions and grown for 4 d at 30 °C.
(961 KB TIF)

Figure S2 Localization of Mis6p-GFP in Wild-Type and Mia1p-Overexpressing Cells The centromere/kinetochore marker, Mis6p-GFP, labeled either two distinct kinetochore clusters (upper panel), similar to wild-type control, or localized to six closely positioned dots, likely representing nonsegregated anaphase kinetochores (lower panel). Shown are single maximum-intensity reconstructions of live cells.
(447 KB TIF)

Figure S3 The Majority of Mia1p-Overexpressing Cells Exhibit Mad2p-GFP on the NE Graph quantifying the proportion of cells with Mad2p-GFP on kinetochores in wild type and cells overexpressing Mia1p (*n* = 100). Inset displays typical appearance of Mad2p-GFP in Mia1p-overexpressing cells, either on kinetochores or broadly spread around the NE. Shown are single maximum-intensity reconstructions of live cells.
(555 KB TIF)

Figure S4 The SPBs Are Displaced from the Spindle Poles in Cells Overexpressing Mia1p (A) Immunofluorescence images of wild-type and Mia1p-overexpressing cells using anti–α-tubulin antibodies, Sad1p-GFP, and the DNA dye, DAPI.
(B) Graph quantifying proportion of mitotic Mia1p-overexpressing cells exhibiting normal or aberrant spindle architecture (*n* = 100).
(996 KB TIF)

Figure S5 The γ-TuRC Complex Protein, Alp4p-GFP, Localizes to Acentrosomal Spindle Poles in Mia1p-Overexpressing Cells Double fluorescence microscopy images of control and Mia1p-overexpressing Alp4p-GFP Sad1p-DSRed cells. Shown are single maximum-intensity reconstructions of live cells.
(489 KB TIF)

Figure S6 Overexpression of Mia1p Induces Abnormal NE Deformations in *pim1–1* Cells at the Permissive Temperature (A) Epifluorescence images of the NE labeled by Uch2p-GFP in wild-type and Mia1p-overexpressing *pim1–1* cells at 24 °C. Shown are single maximum-intensity reconstructions of live cells.
(B) Graph representing the proportion of cells forming the panhandle-shaped protrusions in both genetic backgrounds.
(298 KB TIF)

Figure S7 Epifluorescence Images of Fixed Mia1p-Overexpressing *pim1–1* Cells Containing the Core SPB Marker, Pcp1p-GFP, and the Outer NE Marker, Uch2p-GFP, at 24 °C Rarely, we could observe Mia1p-overexpressing *pim1–1* cells with acentrosomal spindles that exhibited two or more closely positioned DNA masses at the permissive temperature. DNA is stained with DAPI. Shown are single maximum-intensity reconstructions of *z*-stacks.
(492 KB TIF)

Table S1 List of S. pombe Strains Used in This Study (49 KB DOC)

Video S1 Time-Lapse Sequence of a Cut11p-GFP–Expressing Wild-Type Cell Undergoing Mitosis Three-dimensional time-lapse images were generated on a Zeiss Axiovert 200M microscope equipped with CSU10 confocal optical scanner, 488-nm laser illumination source, and CoolSnap digital camera. Image stacks consisted of six sections at 0.5-μm spacing taken every 30 s. Maximum-intensity projection images of each stack were combined into a QuickTime video.
(584 KB MOV)

Video S2 Time-Lapse Sequence of a Mia1p-Overexpressing Cut11p-GFP Cell Undergoing Mitosis Three-dimensional time-lapse images were generated on a Zeiss Axiovert 200M microscope equipped with CSU10 confocal optical scanner, 488-nm laser illumination source, and CoolSnap digital camera. Image stacks consisted of six sections at 0.5-μm spacing taken every 30 s. Maximum-intensity projection images of each stack were combined into a QuickTime video.
(353 KB MOV)

Video S3 QuickTime Video Was Assembled Based on Electron Microscopy Data Presented in Figure 3
Serial sections were aligned and modeled using the IMOD software package. The NE is in green, the SPBs are in purple, and microtubules are in yellow.
(1.4 MB MOV)

Video S4 Time-Lapse Sequence of an Uch2p-GFP α-Tubulin-GFP–Expressing *pim1–1* Cell Undergoing Anaphase at the Permissive Temperature of 24 °C Three-dimensional time-lapse images were generated on a Zeiss Axiovert 200M microscope in epifluorescence mode. Image stacks consisted of five sections at 0.5-μm spacing taken every 60 s. Maximum-intensity projection images of each stack were combined into a QuickTime video.
(126 KB MOV)

Video S5 Time-Lapse Sequence of an Uch2p-GFP α-Tubulin-GFP–Expressing *pim1–1* Cell Undergoing Anaphase at the Restrictive Temperature of 36 °C Three-dimensional time-lapse images were generated on a Zeiss Axiovert 200M microscope in epifluorescence mode. Image stacks consisted of five sections at 0.5-μm spacing taken every 60 s. Maximum-intensity projection images of each stack were combined into a QuickTime video.
(618 KB MOV)

### Accession Numbers

The National Center for Biotechnology Information (NCBI) (http://www.ncbi.nlm.nih.gov) accession numbers for the proteins discussed in this paper are Alp4p (BAA77269), Ark1p (O59790), Ase1p (CAC21482), Cdc7p (CAA55382), Clp1p (CAB76271), Cut11p (CAB11272), Cut7p (CAA40738), Dis1p (CAA19278), Mad2p (CAA16846), Mia1p (Q9URY2), Mis6p (BAA20458), Pcp1p (CAB03608), Pim1p (CAB60670), Rlc1p (CAB54151), Sad1p (BAA87133), and Uch2p (CAB52608).

## Abbreviations

GFP: green fluorescent protein
NE: nuclear envelope
SPB: spindle pole body
TACC: transforming acid coiled coil
γ-TuRC: γ-tubulin ring complex
