# Supplementary material for: The Spindle Pole Bodies Facilitate Nuclear Envelope Division during Closed Mitosis in Fission Yeast
Source: PLoS Biol. 2007 Jun 19;5(7):e170. doi: 10.1371/journal.pbio.0050170 (PMC1892572; doi:10.1371/journal.pbio.0050170)
Supplement: Supplementary file 1 [file pbio.0050170.st001.doc]

| SO11 | pREP1-Mia1p / *ade6-210 ura4-D18 leu1-32* | This study |
| --- | --- | --- |
| SO34 | pREP1-Mia1p / Clp1p-GFP::KanR *ura4-D18 leu1-32 ade6M-2X* | This study |
| SO87 | pREP1-Mia1p / Rlc1p-GFP::ura *ura4-D18 leu1-32 ade6M-2X* | This study |
| SO115 | pREP1-Mia1p / Uch2p-GFP::ura4+ *ura4-D18 leu1-32 ade6M-2X* | This study |
| SO116 | pREP1-Mia1p / Cut11p-GFP::ura *ura4-D18 leu1-32 ade6M-2X* | This study |
| SO197 | pREP1-Mia1p / Uch2p-GFP::ura *pim1-1* *ura4-D18 leu1-32* | This study |
| SO201 | pREP1-Mia1p / LacO(lys1+)Dis1promoterLacI-GFP-NLS(his7+) *leu1-32 ura4-D18* | This study |
| SO502 | pREP1-α-tubulin-GFP / Uch2p-GFP::ura4 *ura4-D18 leu1-32 ade6M-2X* | This study |
| SO1760 | pREP1-α-tubulin-GFP / Uch2p-GFP::ura4  *pim1-1* *ura4-D18 leu1-32* | This study |
| SO1762 | pREP1-α-tubulin-GFP / Cut11p-GFP::ura4 *ade6-704* *leu1-32 ura4-D18* | This study |
| SO1952 | pREP1-Mia1p / Sad1p-GFP::ura4 *ura4-D18 leu1-32 ade6M-2X* | This study |
| SO2002 | pREP1-Mia1p/ Uch2p-GFP::ura4+ Sad1DSRed::KanR *ura4-D18 leu1-32* | This study |
| SO2037 | pREP1-Mia1p / *ase1*Δ::ura *ura4-D18 leu1-32 ade6-216* | This study |
| SO2069 | pREP1-Mia1p / Pcp1p-GFP::kanR Uch2p-GFP:ura4+ *pim1-1 ura4-D18 leu1-32* | This study |
| SO2090 | pREP1-Mia1p / Cut7p-GFP::ura *ura4-D18 leu1-32 ade6M-2X* | This study |
| SO2098 | pREP1-Mia1p / Ase1p-GFP::ura *ura4-D18 leu1-32 ade6M-2X* | This study |
| SO2111 | pREP1-Mia1p / Mad2p-GFP::ura *ura4-D18 leu1-32 ade6M-2X* | This study |
| SO2131 | pREP1-Mia1p / Pcp1p-GFP::KanR *leu1-32* | This study |
| SO2244 | pREP1 / *ura4-D18 leu1-32 ade6-210* | This study |
| SO2318 | pREP1-α-tubulin-GFP / Uch2p-GFP::ura4 *dis1*Δ::KanR *leu1-32 ura4-D18* | This study |
| SO2323 | pREP1-Mia1p / Mis6p-GFP::ura *leu1-32 ura4-D18* | This study |
| SO2341 | pREP1-Mia1p / nmt81-atb2-GFP::KanR Uch2-GFP::ura *leu1-32* | This study |
| SO2363 | pREP1-Mia1p /alp4p-GFP::ura Sad1p-Dsred::KanR *leu1-32 ura4-D18* | This study |
| SO2379 | pREP1-Mia1p / Dis1Cen1GFP Sad1p-Dsred::kanR *pim1-1 leu1-32* | This study |
| SO2440 | pREP1-Mia1p / ark1p-GFP::ura Cut7p-mCherry::ura *leu1-32 ura4-D18* | This study |
| SO2434 | pREP1-Mia1p / alp4p-GFP::ura Cut7p-mCherry::ura *leu1-32 ura4-D18* | This study |
| SO2444 | pREP1-Mia1p / Cut7p-mCherry Pcp1p-GFP::kanR Uch2p-GFP::ura *leu1-32* | This study |
